# Age- and Sex-Specific TSH Upper-Limit Reference Intervals in the General French Population: There Is a Need to Adjust Our Actual Practices

**DOI:** 10.3390/jcm9030792

**Published:** 2020-03-14

**Authors:** Véronique Raverot, Maxime Bonjour, Juliette Abeillon du Payrat, Pauline Perrin, Florence Roucher-Boulez, Helene Lasolle, Fabien Subtil, Françoise Borson-Chazot

**Affiliations:** 1 Hospices Civils de Lyon, LBMMS, Service de Biochimie, Laboratoire d’Hormonologie, Groupement hospitalier Est, F-69677 Bron cedex, France; pauline.perrin03@chu-lyon.fr (P.P.); florence.roucher@chu-lyon.fr (F.R.-B.); 2 Hospices Civils de Lyon, Service de Biostatistique, F-69003 Lyon, France; maxime.bonjour@chu-lyon.fr (M.B.); fabien.subtil@chu-lyon.fr (F.S.); 3 Hospices Civils de Lyon, Fédération d’Endocrinologie, Groupement Hospitalier Est, F-69677 Bron cedex, France; juliette.abeillon@chu-lyon.fr (J.A.d.P.); helene.lasolle@chu-lyon.fr (H.L.); francoise.borson-chazot@chu-lyon.fr (F.B.-C.); 4 Univ Lyon, Université Claude Bernard Lyon 1, Faculté de Médecine Lyon Est, F-69008 Lyon, France

**Keywords:** TSH, elderly, sex, age, reference intervals, hypothyroidism

## Abstract

It is well known that thyroid dysfunction increases with age. This study is aimed to determine reference intervals, in males and females, suitable for thyroid disease exploration during adult life using routinely collected serum thyrotropin (TSH) data in a tertiary center from 2007 to 2018. Over 11 years, 295,775 TSH levels were measured in a single lab. Among the 156,025 TSH results available for analysis, 90,538 values were from female subjects, 82,019 were from patients aged >60 years and 26,825 were from patients aged >80 years. By using an indirect approach, we determined reference values of TSH adapted to age and sex, and we then evaluated the proportion of patients who would have been reclassified with these reference values. The median TSH ranged from 1.2–1.4 mUI/L during the study period. The upper limit of reference range of TSH increased with age; in females the median to 97.5th percentile values increased continuously from the age of 30 years to the oldest age group. Using new calculated reference values in patients with TSH above the conventional upper-limit reference value (4 mUI/L), the proportion of results reclassified as within the reference interval among patients aged >60 years ranged, according to age group, from 50.5% to 65.1% of females and from 33.0% to 37.7% of males. The use of TSH age-specific and sex-specific upper-limit reference values led to the reclassification of a great number of samples, notably among women. This suggests that age-specific TSH upper-limit reference intervals in daily practice should be used in order to avoid misclassification.

## 1. Introduction

The increasing age of the general population represents a new challenge for endocrinologists and caregivers, since it is well known that the occurrence of thyroid dysfunction increases with age and is mostly present in females [1,2,3,4,5,6]. However, although serum thyrotropin (TSH) is the cornerstone of thyroid dysfunction diagnosis, a physiological increase in TSH levels according to age has been reported in many studies worldwide [2,3]. These studies include two European studies that were conducted in Scotland [5] and in the Netherlands [7], but data for the elderly European population remain sparse.

A major question deriving from the change of TSH with age is to decide whether or not TSH reference intervals need to be adapted to the most elderly in order to evaluate their thyroid function. This is of importance, as the proportion of patients who will be classified as having subclinical hypothyroidism may greatly differ according to the upper value of the reference interval [8]. Two different approaches exist to define reference intervals: the direct approach is based on the recruitment and selection of control subjects [9], whereas the indirect approach is based on the analysis of available data from routine testing [10]. The latter has many advantages. For instance, it is based on available data, with no need to collect blood specimens. The data are obtained under real-life conditions and often available in large numbers. In some particular conditions (i.e., among the elderly), criteria that identify a healthy population to define reference values for thyroid exploration cannot be met. Furthermore, the International Federation of Clinical Chemistry (IFCC) has published recommendations to encourage the use of indirect methods to define reference interval [10]. This has been explored by Katayev et al., who reported that a TSH reference interval obtained from “big data” led to very similar results compared to findings from a healthy population tested for antithyroid antibodies [11]. They concluded that estimation of reference interval using stored test results is accurate and reproducible.

The objective of the present study was therefore to determine TSH reference intervals adapted for thyroid disease exploration and follow-up during adult life using the indirect approach and then to evaluate the proportion of the elderly who would have been reclassified with these new reference values.

## 2. Experimental Section

Selection of samples: The data used stem from a single biochemistry laboratory in a tertiary center (Hospices Civils de Lyon) recruiting samples from inpatients and outpatients attending the different sites of the university hospital. All TSH tests performed between March 2007 and December 2018 on samples from patients aged ≥20 years were included. The TSH test was performed using the Architect i2000 immunoassay analyzer (Abbott Laboratories, Abbott Park, IL, USA) with no change of reagent over the 11-year period. The intra-assay coefficient of variation was <3%, and the inter-assay coefficient of variation was <5%. Samples for which the TSH value, the sex or the date of collection were missing were excluded. In order to avoid an over-representation of patients with prior evidence of thyroid disease, we excluded samples with a TSH value <0.1 or >10 mUI/L, samples from the nuclear medicine thyroid unit and samples taken within less than one year for an individual patient. The data were anonymized before analysis. The study was approved by the ethics committee of the institution (Comité d’Éthique du CHU de Lyon; ref 19–155).

Statistics: Distributions of TSH were described separately by 10-year age group and by sex. For the determination of the reference intervals, a parametric probability distribution for TSH values was selected for each age and sex category in order to obtain more precise TSH reference values than using raw quantiles. These distributions were selected between the gamma, the generalized gamma, the log-normal or the Gumbel distribution according to best fit. A Markov chain Monte Carlo (MCMC) approach with noninformative priors was used to estimate the parameters of the distributions, then to calculate the desired quantiles and their 95% confidence interval. The reference intervals were defined by the 2.5% and 97.5% percentiles of the modeled distribution for each group. Other quantiles were also calculated for descriptive purposes.

## 3. Results

**Figure 1:**
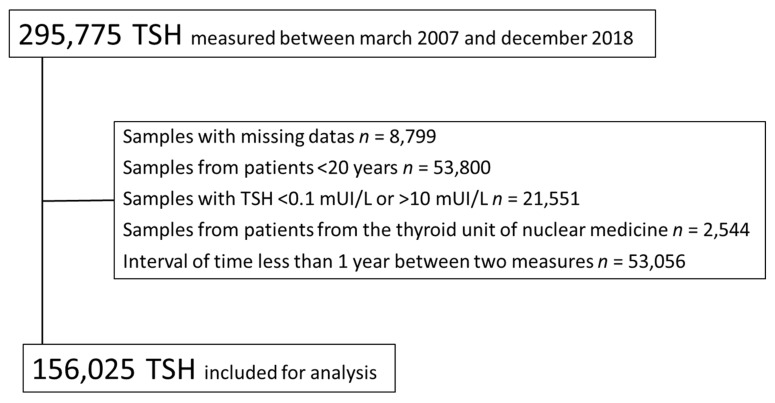
Study flow chart.

**Figure 2:**
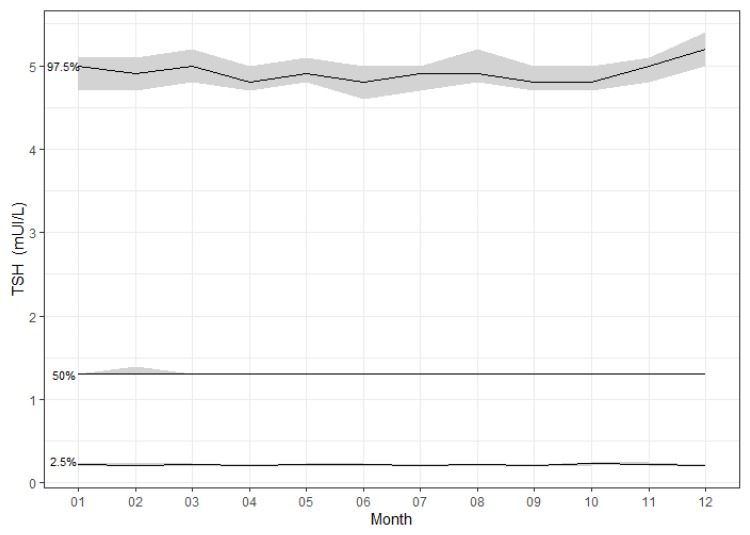
The 2.5th, 50th and 97.5th raw percentiles of thyrotropin (TSH) observed over a year. The grey area corresponds to the 95% confidence intervals of the quantiles.

During the study period, TSH was measured in a total of 295,775 samples. Among these, 156,025 samples were included for analysis (Figure 1), corresponding to 115,794 subjects. There were 90,538 values for female subjects and 65,487 values for male subjects. The median age was 62 years (range: 20–108 years). The number of samples per age group ranged from 4662–28,104 (Table 1). Overall, 82,019 samples (53%) were obtained from patients >60 years of age, 26,825 (17%) from patients >80 years of age, and 4662 (3%) from patients >90 years of age. Over the 11 years of the study, the median TSH ranged from 1.2–1.4 mUI/L. The circannual range of the 97.5th TSH percentile was 4.6–5.4 mUI/L (Figure 2).

**Figure 3:**
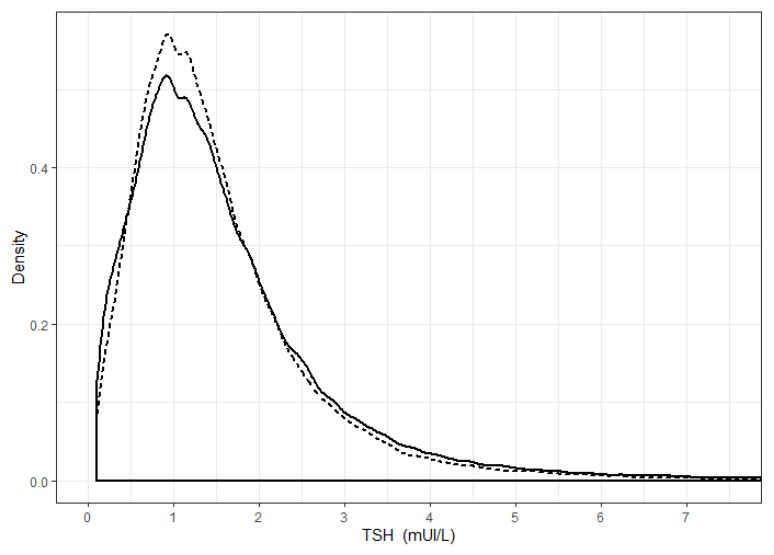
Thyrotropin (TSH) distribution, according to sex. Continuous line: data obtained from females; dotted line: data obtained from males.

The distribution of TSH values was not Gaussian and significantly skewed towards higher TSH values. In females, the median TSH was 1.30 mUI/L (interquartile range, IQR (0.81–2.00)), and in males, it was 1.30 mUI/L (IQR (0.83–1.90); Figure 3).

### Modeling According to Age Group

The results hereafter were obtained from the modeled TSH distributions. Generalized gamma distributions were retained for all age- and sex-groups as they provided the best fit to these data.

**Figure 4:**
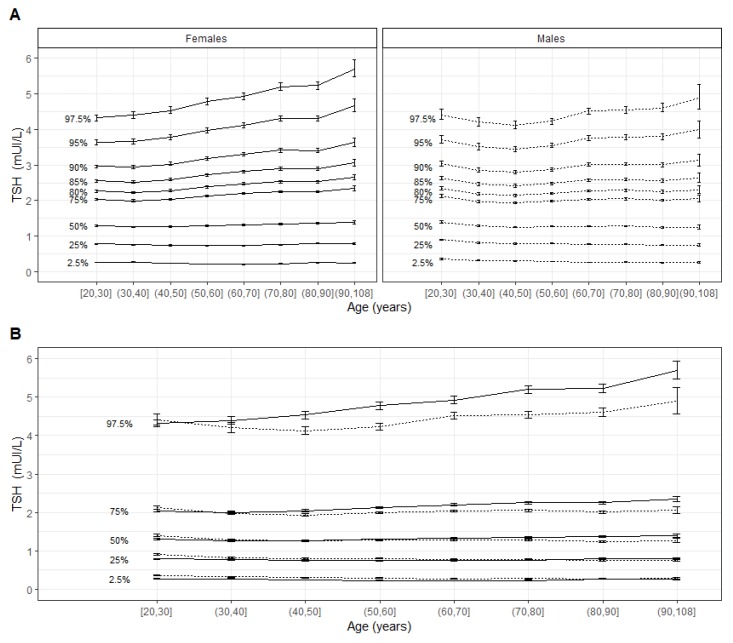
Modeled thyrotropin (TSH) distribution by age group and sex. (**A**) Modeled TSH distribution according to age group in both sexes. (**B**) Modeled TSH distribution according to age group and sex. Values for females are presented as a continuous line and for males as a dotted line.

In females, the value of the TSH percentile increased continuously as a function of age after 30 years from the median to 97.5th percentile. In males, TSH values were not different between age groups for the 2.5th–85th percentiles; there was a slight increase after 40 years of age for higher percentiles (Figure 4A).

The median TSH value and the first quartile for females and males were close for each age group. The third quartile was higher in women compared to men in all age groups after 30 years of age (Figure 4B). The increase according to age of the 97.5th percentile was more pronounced in females than in males (Figure 4B).

**Figure 5:**
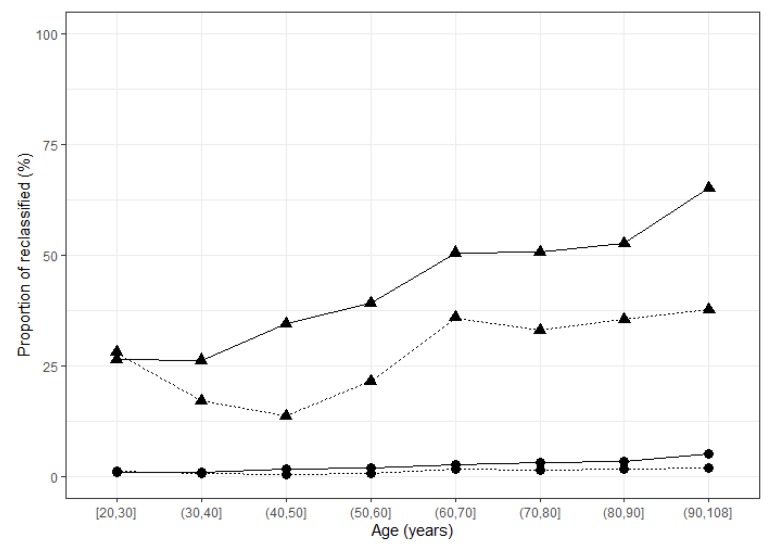
Proportion of patients reclassified according to age group and sex. Solid lines: females; dotted lines: males. Circles: proportion calculated among the total number of samples in the age group; triangles: proportion calculated among samples >4 mUI/L within the age group.

After 30 years of age, the interval was larger in females, mainly because of the higher 97.5th percentile value of TSH (Figure 4B, Table 1). When using the conventional upper-limit reference value (4 mUI/L) on the entire population, 4.7% of the TSH values were above the upper limit (5.2% in females and 4.0% in males). According to the age group, this proportion ranged from 3.7–7.9% in females and 3.2–4.9% in males (Table 2). Among the values that were above the conventional upper-limit reference value, 26.3–65.1% and 13.6–37.7% were reclassified as within the reference interval in females and in males, respectively (Figure 5, Table 2). In females and males >60 years of age, 50.5–65.1% of TSH results considered as elevated when using the conventional TSH upper value of 4 mUI/L were reclassified within the reference range when using new calculated reference intervals; this same range of results was 33.0–37.7% in males >60 years of age.

## 4. Discussion

In the present study, we found that, while the median TSH did not change in function of age, the upper limit of the reference range did, and this was more pronounced in females. We established new reference values and found that more than half of women >60 years of age would have been reclassified from an elevated to a within reference interval TSH level using age-adapted reference values.

Our study has two major strengths. The first strength was the sample size, which was very large and included a high number of patients aged >80 years. Second, the TSH assay was performed in a single laboratory using the same reagent during the whole study period, and we did not find any clinically relevant change in median TSH levels over the length of the study. One of the limitations is the lack of thyroid antibody test results. Thus, it cannot be excluded that some patients had potential thyroid illness. However, a number of studies have shown that TSH levels are not impacted by thyroid antibody status, suggesting that it was not an important limiting factor. For instance, Vadiveloo et al. concluded that the distribution of TSH values shifted towards a higher concentration as a function of age regardless of the presence or absence of thyroid antibodies [5]. This was also reported by Takeda et al. [12]. Another limitation of the present study is that patients were not followed over time, but published longitudinal studies have reported that the increase in TSH over time was limited [3]. In particular, Waring et al. noted that, over 13 years, <7% of their cohort had an increase of TSH >2 mUI/L, 20% an increase >1 mUI/L, and 80% an increase ≤1 mUI/L [13]. It is also of note that samples with a TSH <0.1 and >10 mUI/L were excluded to avoid an over-representation of patients with a priori evidence of thyroid disease. However, samples could have been from patients with antithyroid antibody (unlikely to affect the conclusions as discussed above) or also from patients receiving thyroid hormones or medicine altering thyroid function.

Similar to other studies, we found higher upper limits of the reference value in females compared to males [2,5,14]. For median TSH values, this was more heterogeneous. The median TSH value was similar to that found in the NHANES III cohort and the study reported by Yoshihara et al. in Japan [2,15], but lower than in studies conducted in Spain and China [16,17]. This might be related to the age of the patients included and also to the iodine status of the population. Indeed, the north of Spain has a normal iodine nutritional status in school-aged children [18], and China is also known to have normal iodine status [19]. Conversely, although Europe is known to be iodine sufficient [20], there is a trend towards iodine insufficiency in pregnant women in the Lyon area [21]. In contrast with these reports, in the Rotterdam Study, the median value decreased in function of age [7]. The median value in the oldest patients was comparable to that found in both the NHANES III and our study, but the median TSH of the youngest patients was much higher [2] than in the present study. More generally, we found that the increase in TSH affected only half of females and 15% of males; however, we were not able to further analyze this potentially interesting feature as the study did not include any clinical data.

Yoshihara et al. [15] found a relationship between TSH and daily temperatures represented by months of the year. They studied TSH values obtained from a single center for six consecutive years and measured with a single analytic method. They found a significant decrease in median TSH during summer months, but in the present study we did not find any change. Similarly, before us, Ehrenkranz et al. did not find any significant variation of TSH distribution with the time of the year [22].

When the new upper reference limits for each age group defined in the present study were applied to the study population, between a quarter and two-thirds of females with an elevated TSH according to the conventional threshold were reclassified as normal. The proportion of men reclassified was lower but reached over a third. It is of note that this proportion seems very high compared to that reported by Kahapola-Arachchige et al. [4]. However, in their study, the authors used the number of patients in each age/sex group and not the number of patients above the conventional threshold (which is more informative as to the size of the effect). The proportion of reclassified values increased with age in both sexes, owing on the one hand to the increasing TSH value according to age and on the other to the definition of age-adapted reference intervals according to distribution. This point is of particular importance since this may avoid unnecessary exams, consultations, and even treatments which are associated with cardiovascular and bone risks in cases of overtreatment in older patients [23]. Due to an increasingly aging population, reconsidering interpreting TSH values according to age and sex may be very useful. Some authors advocate not initiating treatment with thyroid hormone unless TSH is above very high values [23]. This is an on-going but controversial debate [24], but regardless of the need to treat or not, current recommendations are against screening for asymptomatic thyroid dysfunction in primary care [25].

## 5. Conclusions

In this large cohort, the use of age- and sex-adapted reference intervals in patients >60 years allowed reclassification of more than half of TSH results in women and a third in men. This suggests that age-specific TSH reference intervals in daily practice should be used in order to avoid misclassification.

## Figures and Tables

**Table 1:** Modeled serum thyrotropin (TSH) according to quantile, sex and age group (reference values are given by 2.5th and 97.5th percentiles).

			TSH (mUI/L) [95%CI]								
			2.50%	25%	50%	75%	80%	85%	90%	95%	97.50%
	Age	*n*									
Female	(20,30)	10,906	0.26 (0.26, 0.27)	0.79 (0.77, 0.80)	1.30 (1.28, 1.32)	2.04 (2.01, 2.07)	2.27 (2.24, 2.30)	2.56 (2.52, 2.59)	2.96 (2.91, 3.00)	3.64 (3.58, 3.71)	4.32 (4.24, 4.41)
	(30,40)	11,824	0.27 (0.26, 0.28)	0.77 (0.75, 0.78)	1.26 (1.24, 1.28)	2.00 (1.97, 2.02)	2.23 (2.19, 2.26)	2.52 (2.48, 2.56)	2.94 (2.89, 2.98)	3.66 (3.60, 3.73)	4.39 (4.31, 4.49)
	(40,50)	10,500	0.24 (0.23, 0.24)	0.74 (0.73, 0.75)	1.26 (1.24, 1.28)	2.04 (2.01, 2.07)	2.28 (2.25, 2.32)	2.59 (2.55, 2.63)	3.03 (2.98, 3.08)	3.78 (3.71, 3.85)	4.53 (4.44, 4.64)
	(50,60)	12,530	0.22 (0.21, 0.23)	0.74 (0.72, 0.75)	1.29 (1.27, 1.31)	2.13 (2.10, 2.15)	2.39 (2.35, 2.42)	2.72 (2.68, 2.76)	3.18 (3.13, 3.23)	3.98 (3.91, 4.05)	4.78 (4.68, 4.87)
	(60,70)	13,846	0.21 (0.20, 0.21)	0.74 (0.73, 0.75)	1.32 (1.30, 1.34)	2.20 (2.17, 2.23)	2.47 (2.44, 2.50)	2.82 (2.78, 2.85)	3.30 (3.25, 3.35)	4.12 (4.05, 4.19)	4.93 (4.84, 5.02)
	(70,80)	13,880	0.22 (0.21, 0.23)	0.76 (0.74, 0.77)	1.34 (1.32, 1.36)	2.25 (2.22, 2.28)	2.53 (2.50, 2.57)	2.90 (2.86, 2.94)	3.41 (3.36, 3.47)	4.31 (4.23, 4.38)	5.20 (5.10, 5.31)
	(80,90)	13,643	0.25 (0.25, 0.26)	0.79 (0.78, 0.81)	1.36 (1.34, 1.38)	2.25 (2.22, 2.28)	2.53 (2.49, 2.56)	2.89 (2.85, 2.93)	3.41 (3.36, 3.46)	4.31 (4.24, 4.39)	5.23 (5.13, 5.34)
	(90,108)	3409	0.25 (0.23, 0.26)	0.79 (0.76, 0.82)	1.39 (1.35, 1.43)	2.34 (2.28, 2.41)	2.65 (2.58, 2.73)	3.06 (2.97, 3.16)	3.64 (3.53, 3.77)	4.66 (4.49, 4.85)	5.71 (5.47, 5.95)
Male	(20,30)	4294	0.36 (0.34, 0.37)	0.90 (0.88, 0.92)	1.40 (1.37, 1.43)	2.13 (2.08, 2.17)	2.35 (2.30, 2.40)	2.63 (2.58, 2.69)	3.03 (2.96, 3.11)	3.72 (3.62, 3.83)	4.42 (4.28, 4.56)
	(30,40)	5119	0.32 (0.31, 0.33)	0.82 (0.80, 0.83)	1.29 (1.26, 1.31)	1.97 (1.94, 2.01)	2.19 (2.15, 2.23)	2.46 (2.41, 2.51)	2.85 (2.79, 2.91)	3.52 (3.43, 3.61)	4.21 (4.08, 4.34)
	(40,50)	7551	0.30 (0.29, 0.31)	0.79 (0.77, 0.80)	1.25 (1.23, 1.27)	1.93 (1.90, 1.97)	2.15 (2.11, 2.18)	2.42 (2.37, 2.46)	2.80 (2.75, 2.85)	3.45 (3.38, 3.53)	4.13 (4.03, 4.23)
	(50,60)	11,282	0.29 (0.28, 0.30)	0.79 (0.78, 0.81)	1.28 (1.26, 1.30)	1.99 (1.96, 2.01)	2.21 (2.18, 2.24)	2.49 (2.45, 2.52)	2.88 (2.84, 2.92)	3.55 (3.49, 3.61)	4.23 (4.15, 4.32)
	(60,70)	14,258	0.26 (0.26, 0.27)	0.77 (0.76, 0.78)	1.28 (1.26, 1.29)	2.04 (2.01, 2.06)	2.28 (2.25, 2.31)	2.58 (2.55, 2.62)	3.01 (2.97, 3.06)	3.76 (3.70, 3.82)	4.51 (4.43, 4.60)
	(70,80)	13,210	0.27 (0.26, 0.28)	0.78 (0.77, 0.79)	1.28 (1.27, 1.30)	2.05 (2.02, 2.07)	2.29 (2.26, 2.32)	2.59 (2.56, 2.63)	3.03 (2.98, 3.07)	3.78 (3.72, 3.85)	4.55 (4.46, 4.64)
	(80,90)	8520	0.26 (0.25, 0.27)	0.75 (0.73, 0.76)	1.24 (1.22, 1.26)	2.01 (1.98, 2.04)	2.25 (2.21, 2.29)	2.56 (2.52, 2.61)	3.01 (2.96, 3.07)	3.80 (3.71, 3.88)	4.61 (4.49, 4.73)
	(90,108)	1253	0.27 (0.24, 0.29)	0.75 (0.72, 0.79)	1.26 (1.20, 1.31)	2.05 (1.96, 2.15)	2.31 (2.20, 2.42)	2.64 (2.52, 2.78)	3.13 (2.97, 3.30)	3.99 (3.76, 4.25)	4.89 (4.57, 5.26)

**Table 2:** Reclassification of samples according to sex and age group.

Sex	Age, Years	N	Tsh Above the Conventional Upper-Limit Reference Value (4 mui/l), *n* (%)	Tsh Above the New Calculated Reference Values, n (%)	Proportion of Reclassified as within the Reference Interval by Using Age-Group Reference Interval Upper Limit, %	
					Compared the Number of Subjects with tsh > 4 mui/l	Compared to Total Number of Subjects in the Group
Female		90,538	4680 (5.2)	2611 (2.9)	44.2	2.3
	(20,30)	10,906	399 (3.7)	294 (2.7)	26.3	1.0
	(30,40)	11,824	465 (3.9)	343 (2.9)	26.2	1.0
	(40,50)	10,500	459 (4.4)	301 (2.9)	34.4	1.5
	(50,60)	12,530	617 (4.9)	376 (3.0)	39.1	1.9
	(60,70)	13,846	749 (5.4)	371 (2.7)	50.5	2.7
	(70,80)	13,880	856 (6.2)	422 (3.0)	50.7	3.1
	(80,90)	13,643	866 (6.3)	410 (3.0)	52.7	3.3
	(90,108)	3409	269 (7.9)	94 (2.8)	65.1	5.1
Male		65,487	2589 (4.0)	1836 (2.8)	29.1	1.1
	(20,30)	4294	168 (3.9)	121 (2.8)	28.0	1.1
	(30,40)	5119	181 (3.5)	150 (2.9)	17.1	0.6
	(40,50)	7551	242 (3.2)	209 (2.8)	13.6	0.4
	(50,60)	11,282	393 (3.5)	309 (2.7)	21.4	0.7
	(60,70)	14,258	598 (4.2)	384 (2.7)	35.8	1.5
	(70,80)	13,210	579 (4.4)	388 (2.9)	33.0	1.4
	(80,90)	8520	367 (4.3)	237 (2.8)	35.4	1.5
	(90,108)	1253	61 (4.9)	38 (3.0)	37.7	1.8
Total population		156,025	7269 (4.7)	4447 (2.9)	38.8	1.8
